# Chemotherapy and Physical Therapeutics Modulate Antigens on Cancer Cells

**DOI:** 10.3389/fimmu.2022.889950

**Published:** 2022-07-06

**Authors:** Wojciech Szlasa, Natalia Janicka, Natalia Sauer, Olga Michel, Bernadetta Nowak, Jolanta Saczko, Julita Kulbacka

**Affiliations:** ^1^ Faculty of Medicine, Wroclaw Medical University, Wroclaw, Poland; ^2^ Faculty of Pharmacy, Wroclaw Medical University, Wroclaw, Poland; ^3^ Department of Molecular and Cellular Biology, Faculty of Pharmacy, Wroclaw Medical University, Wroclaw, Poland

**Keywords:** antigens, membrane, modulation, anticancer therapy, cancer

## Abstract

Cancer cells possess specific properties, such as multidrug resistance or unlimited proliferation potential, due to the presence of specific proteins on their cell membranes. The release of proliferation-related proteins from the membrane can evoke a loss of adaptive ability in cancer cells and thus enhance the effects of anticancer therapy. The upregulation of cancer-specific membrane antigens results in a better outcome of immunotherapy. Moreover, cytotoxic T-cells may also become more effective when stimulated *ex-vivo* toward the anticancer response. Therefore, the modulation of membrane proteins may serve as an interesting attempt in anticancer therapy. The presence of membrane antigens relies on various physical factors such as temperature, exposure to radiation, or drugs. Therefore, changing the tumor microenvironment conditions may lead to cancer cells becoming sensitized to subsequent therapy. This paper focuses on the therapeutic approaches modulating membrane antigens and enzymes in anticancer therapy. It aims to analyze the possible methods for modulating the antigens, such as pharmacological treatment, electric field treatment, photodynamic reaction, treatment with magnetic field or X-ray radiation. Besides, an overview of the effects of chemotherapy and immunotherapy on the immunophenotype of cancer cells is presented. Finally, the authors review the clinical trials that involved the modulation of cell immunophenotype in anticancer therapy.

**Graphical Abstract:**
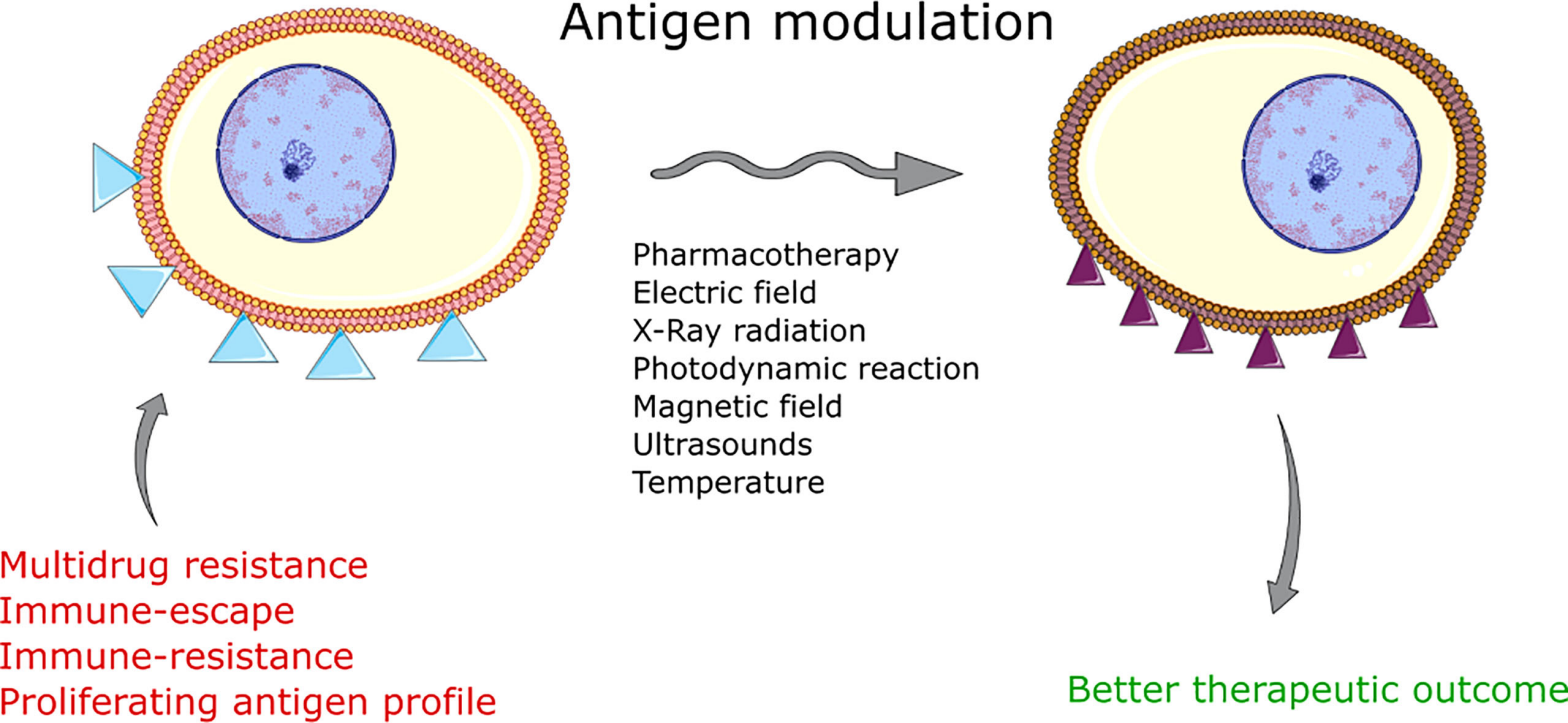


## Introduction

‘Membrane antigens’ is a term used by researchers to refer to the molecules present on the cell membrane that may be recognized by immune system cells. Modulation of membrane components remains a unique approach in anticancer therapy (1). By the sensitization of cancer, scientists aim to overexpress tumor-specific molecules found on cell membranes (2–6). This method attempts to overcome the immune escape of cancer cells and aims to make single cancer cells visible to the immune system. The controlled regulation of the immunophenotype of cells can overexpress or downregulate the expression of specific plasma membrane-associated antigens (7, 8). The method opens a new perspective for sensitizing the cells to the subsequent standard therapy and expecting a better clinical outcome (5).

Immunotherapy focuses on targeting cancer-related antigens on the cell surface (4). The more an antigen is specific to cancer, the fewer side effects occur. For instance, when targeting CD20 in leukaemia therapy, it is blood and bone marrow that are most affected (9). However, when MAGE receptors are targeted in melanoma therapy, testes and blood are affected as well (10). The efficacy of the treatment also depends on the abundance of the targeted antigen on cancer cells. Specifically, the more targets, the higher the probability of recognizing cancer cells by antibodies or T-cell receptors (TCR) (11).

Tumor immunotherapy may be based on cellular therapeutics or typical immunotherapy agents such as antibodies (12, 13). Both options rely on targeting tumor cells by binding to a specific epitope. When a specific antigen is targeted with an antibody, the cell with the attached antibody might become an ideal target for immune cells (14). Moreover, the antibody-antigen interaction alone may reduce the activity of the targeted protein or even decrease its enzymatic activity (15). Sometimes the antibody remains conjugated with the radioisotope or chemotherapy agent (16, 17). In such cases, the cytotoxic effect is stronger and more specific than in systemic therapy. Conversely, when the reaction with the antigen depends on the interaction of TCR or membrane-bound immunoglobulin with the antigen, then the whole T-cell is involved in the interaction with the target (18). The abundance of the target of immunotherapy affects the immune recognition of the alien cell (19).

There are at least three mechanisms that affect the targeting of cancer cells. The first one involves the loss of human leukocyte antigen (HLA) in the least differentiated cancers. Here, the alien cell should be eliminated by NK cells due to the lack of the major histocompatibility complex (MHC) I molecules in the missing-self mechanism (20). However, in some cases, NK cells might not kill a cell lacking MHC (21). The second factor is the presence of cancer-specific molecules that may become targets of the therapy (22). An ideal target should be specific to cancer and highly abundant on cancer cells. However, tumors differ in the expression of cancer-specific molecules (23–27). In general, high expression of cancer antigen is required for effective therapy and may be effectively treated with targeted immunotherapy (19). The third option – the PD-1 and CTLA-4 molecule pathways show that the effects of the expression of specific target molecules might be depleted by the immune escape of the cancer cell (28). The cell induces the expression of molecules that prevent/inhibit the activation of the lymphocyte and thus anticancer activity. Interestingly, some studies showed an attempt to target the tumor microenvironment, even presenting the advantages of this method over the blockade of immune checkpoints in the tumor (29, 30).

The paper reviews various methods for the regulation of membrane antigens. First, it presents an overview of the effects of chemotherapy on immunotherapy-related antigens on cancer cells. In the next paragraphs, electroporation, photodynamic therapy, x-ray radiation, and the application of ultrasounds or magnetic fields are reviewed in case of the modulatory effects on membrane antigens. Finally, the authors present the clinical trials involving the modulation of membrane antigens in anticancer therapy.

### Methods for Modulation of Membrane Antigens

#### Chemotherapy and Pharmacotherapy

Chemotherapy may modulate the expression of membrane antigens *via* several distinct pathways. First, some drugs can interact with transcription factors, directly affecting the expression of the protein (31). Second, chemotherapy agents might interact with the already synthesized protein. The interaction can inhibit or stimulate the protein’s function (32, 33). When a chemotherapy drug enhances the activity of the membrane protein, no additional units of the protein are required. Conversely, when the interaction remains inhibitory or the ligand blocks the receptor’s binding pocket and when units of a new protein are required for cell functioning, the new protein may be biosynthesized, and thus the overall expression of the protein may increase (34). However, when a negative interaction between ligand and the receptor occurs, sometimes no additional protein is synthesized. For instance, when the chemotherapy agent blocks the receptor, which transmits the biological signal, the cell might shift toward the alternative signaling pathway and then the ligand-receptor interaction does not affect the expression of the protein (35).

#### SERMs

The first group of chemotherapy drugs that modulate the expression of hormone receptors on cells is hormone agonists or antagonists. Hormone agonists, *via* binding to the receptor, may stimulate the receptor and transduce the hormonal signal to the nucleus (36). On the other hand, the binding of the agonist might lead to the blockage of the receptor, and therefore, the hormones cannot act at the cellular level. Overexpression of the receptor may overcome the blockage of the receptor and result in the cell becoming sensitive to hormonal stimulation. Such tendencies are mostly observed in prostate and breast cancers (37–39). The latter is connected with the expression of estrogen receptor which is a predictive and prognostic marker in hormone-dependent cancers.

Selective estrogen receptor modulators (SERMs) are commonly used in breast tumors expressing estrogen receptor-α (ERα) (40). One of them, tamoxifen, is a prodrug metabolized by the cytochrome P450 system to 4-hydroxytamoxifen – a pharmacologically active molecule. The estrogen receptor consists of two subunits: α (ERα) and β (ERβ) and their proportions depend on the tissue. ERα plays a significant role in the development of the mammary gland, bone mineral density and the hypothalamic-pituitary axis, while ERβ, among others, regulates ovulation. The ligand (estrogen) binds to the ER and leads to receptor activation, next step is the recruitment of coactivators or corepressors involved in the transcription process. The ligand-receptor complex ultimately activates or inhibits the production of a specific protein by the cell (41). In breast cancer, estrogens stimulating ERα increase proliferation, while activating ERβ inhibits the ability to reproduce. Reduced proliferation is associated with the activation or inhibition of the expression of genes regulated by estrogen receptors and the modulation of cell signaling pathways (42, 43). A study by Saji et al. suggests that the presence of ERβ is beneficial in the hormonal treatment of ER-positive breast cancer (44). Long-term blockade of the estrogen receptor by SERMs (especially tamoxifen) results in the development of resistance that may also engage cross-talk pathways. It leads to increased ER expression in breast cancer cells associated with receptor activation and hypersensitivity to low estrogen levels (45). Overexpression of membrane antigens such as ERBB2 (HER2/neu) and/or epidermal growth factor receptor (EGFR/HER1) is associated with a significant reduction of response to treatment with tamoxifen but greater sensitivity to letrozole (46). Long-term stimulation of estrogen receptors in the endometrium may induce proliferation and increase the risk of uterine cancer (41).

The expression of ERβ in cancer may lead to the inhibition of tumor growth (47). For this reason, ERβ agonists may find application in the prevention or treatment of colorectal cancer in which the levels of these receptors are reduced compared to normal expression in the colorectal epithelium (42, 48). Prinaberel (ERB-041, strong and selective ERβ stimulator) inhibits proliferation, increases tumor cell death by modulating the expression of specific genes (49). Studies show that CXC motif chemokine receptor 4 (CXCR4) expression is decreased during treatment with two selective ERβ agonists: ERB-041 and liquiritigenin (47). CXCR4 is a transmembrane protein and, together with ligand stromal cell-derived factor-1 (SDF-1) is relevant in the invasion of cancer cells (50). Stimulation of the β subunit of the estrogen receptor has the same effect in prostate cancer (51). Therefore, tamoxifen and raloxifene reduce cell migration, prevent the development of prostate cancer and the formation of metastases (52). It is worth noting that cell death in prostate cancer occurs as a result of androgen-independent apoptosis connected with the activation of ERβ (51). The activation of ERβ by a selective ERβ agonist diarylpropionitrile (DPN) increases the adhesion of breast cancer cells by enhancing the surface expression of integrin α1 and integrin β1 (53). The effect also reduces the migration of neoplastic cells (53). ERb1 affects the up-regulation of E-cadherin expression by inhibiting its transcriptional repressors ZEB1/2 and up-regulating miR-200a, miR-200b and miR-429 in basal-like breast cancer (54). Cadherins are adhesion proteins that are important for maintaining tight cell connections. Stimulation of the β subunit of the estrogen receptor in androgen-independent prostate cancer cells leads to the modulation of the expression of adhesion proteins. DPN downregulates N-cadherin expression in PC-3 cells and increases the expression of E-cadherin and β-catenin in the cell membrane of the DU-145 line (55). This promising result prompted a search for more ERβ agonists which may have important therapeutic significance. Besides, ERβ downregulates EGFR transcription (56).

Estrogen receptor antagonists are effective drugs in hormone-dependent breast cancer. One of them, fulvestrant (ICI 182,780) is a pure antagonist with a high binding affinity, devoid of agonist activity in other tissues as compared to SERMs. As a result of impaired dimerization, the receptor cannot stimulate or inhibit the transcription of target genes (57). It inhibits the nuclear transport of ER and contributes to proteasome-mediated ER destruction, therefore it is classified as a selective estrogen receptor degrader (SERD) (58, 59). The advantage of fulvestrant over tamoxifen is that it reduces the level of estrogen receptors (ER) and progesterone receptors in cancer cells (57). Fulvestrant is indicated especially in locally advanced or metastatic hormone receptor-positive (HR+) and human epidermal growth factor 2 negative (HER2-) breast cancer in postmenopausal women (60, 61).

#### Aromatase Inhibitors

Aromatase inhibitors (AI) are essential in treating ER-positive breast cancer in women after menopause. Aromatase is an enzyme that is involved in the final step in steroid biosynthesis, which is the conversion of androgens into estrogens (62). Third-generation AI are divided into steroidal or type I (structural similarity to androstenedione – a natural ligand) and non-steroidal (type II) aromatase inhibitors. Type I steroidal drugs include formestane and exemestane. Exemestane binds directly to the androstenedione binding site and consequently has an irreversible inhibitory effect. It competes with androstenedione and testosterone (63), reducing estrogen levels, which inhibits hormone-stimulated breast cancer cells. Exemestane exhibits modest androgenic activity, which may prevent increased bone loss compared to non-steroidal aromatase inhibitors (64). Anastrozole and letrozole are classified as nonsteroidal type II inhibitors. They effectively and reversibly inhibit the activity of aromatase. Meta-analyses of phase 3 randomized controlled trials prove that with aromatase inhibitors, progression-free survival (PFS) is longer, although no significant change in overall survival in postmenopausal women with HR+ advanced breast cancer has been noted (65).

Aromatase inhibitors are considered effective second-line agents following the occurrence of tamoxifen-induced drug resistance (66). However, breast cancer cells may also exhibit resistance to aromatase inhibitors. Drug-induced estrogen deprivation results in hypersensitivity of estrogen receptors to residual estrogen levels. One of the reasons for the decreased response to treatment is the adaptive increase in the expression of estrogen receptors. Stimulation of ER is also connected with the intensified expression of ERBB2 (HER2/neu)/ERBB3, mitogen-activated protein kinases (MAPKs) and insulin-like growth factor IGF-receptor signaling caused by long-term estrogen deprivation (LTED) (45). ERs are mainly nuclear receptors, but it is worth noting that they are also present in the cytoplasm and cell membrane, because they originate from the same transcript (67). Extranuclear ER after binding estrogen interacts with transmembrane EGFR and insulin-like growth factor-I receptor (IGF-IR) to initiate signaling for cell proliferation (68). As a result of activation of the membrane receptor, neoplastic cells will proliferate and increase the tumor mass (68).

#### Androgen Deprivation Therapy

Androgen deprivation therapy (ADT) can lead to the modulation of prostate-specific membrane antigen (PSMA) levels in prostate cancer patients. The reason is the use of drugs that reduce the concentration of androgens, including luteinizing hormone-releasing hormone (LHRH) agonists, such as leuprolide or goserelin LHRH antagonists, for example, degarelix. Androgen receptor (AR) inhibitors such as bicalutamide, flutamide or enzalutamide, and androgen synthesis inhibitors – abiraterone, also cause androgen deprivation. Previous studies suggest that short-term ADT increases PSMA expression, while long-term ADT has the opposite effect (7, 8, 69). The up-regulation of prostate-specific membrane antigen expression may be an attractive target of therapy in the future. Phase 1 of the clinical trial proves that an antibody-drug conjugate (ADC) targeting PSMA has specific anti-tumor activity not only in the preclinical model. PSMA ADC consists of a fully human monoclonal IgG1 antibody conjugated to monomethyl auristatin E (MMAE) through a dipeptide linker (valine-citrulline) that disintegrates inside the tumor cell. MMAE selectively binds to PSMA-positive cells, it inhibits microtubule polymerization, resulting in cell cycle arrest and cell death (70). The quick internalization of membrane antigen improves the transport of the conjugate into the cell (71).

#### Standard Chemotherapy Agents and Natural Compounds

Docetaxel increases surface expression of the carcinoembryonic antigen (CEA), calreticulin (CRT), mucin-1 (MUC-1) and Fas in cancer cells. CRT is expressed on the surface of dying cells, which allows dendritic cells to present antigens. A higher concentration of CRT enables a faster and more effective response of the immune system (72). Docetaxel-resistant cells also showed elevated levels of Fas or CEA and were lysed by cytotoxic T lymphocytes (CTL) after exposure to docetaxel. This proves that cancer cells, despite not responding to this drug, are immunogenically modulated. In the case of prostate cancer in humans, the presence of docetaxel resulted in increased levels of prostate-specific antigen (PSA), prostate stem cell antigen (PSCA) and PSMA. This drug leads to high sensitivity to antigen-specific CTL killing (1).

**Figure 1:**
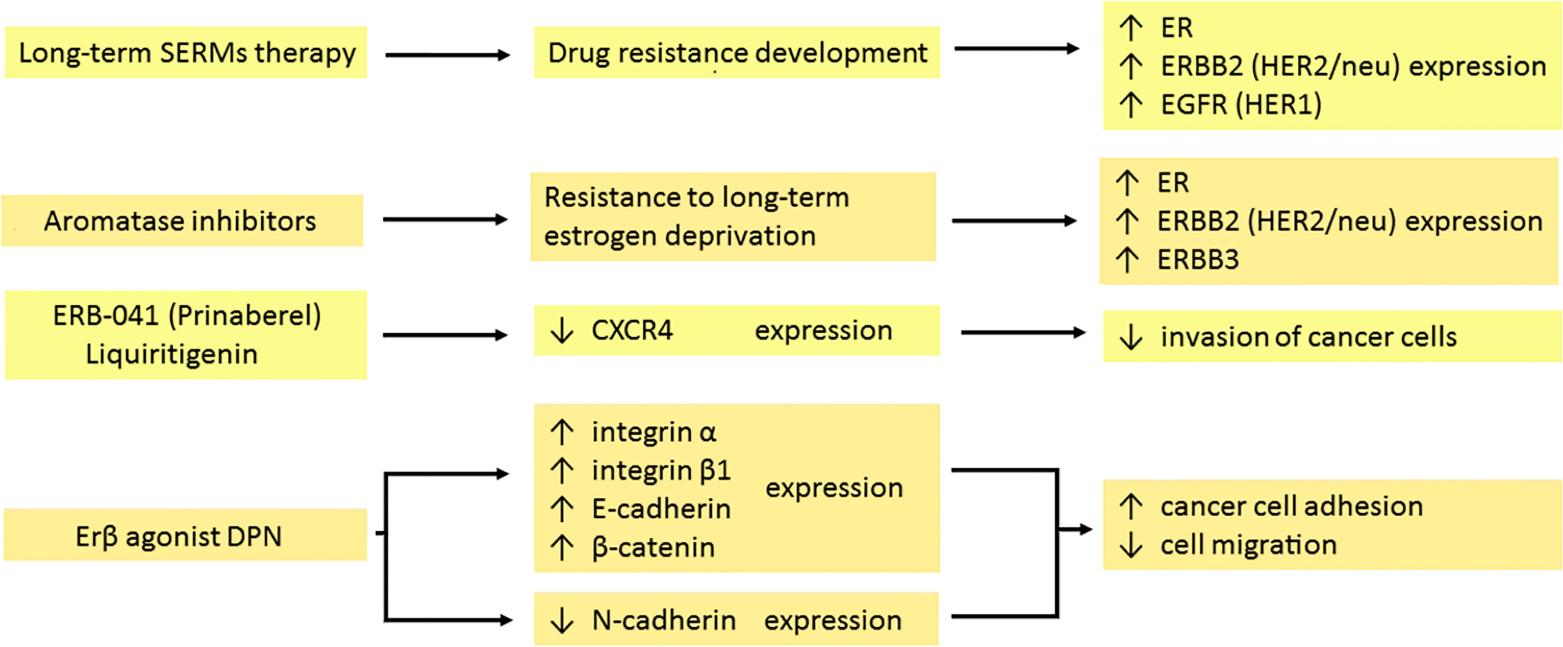
Effects of hormonal therapy of the tumour on the expression of membrane antigens. Long-term therapy with both selective estrogen receptor modulators (SERMs) and aromatase inhibitors (AI) leads to the development of drug resistance. In response, cancer cells increase the expression of several receptors: ER, ERBB2 (HER2/neu), ERBB3 and EGFR (HER1). Prinaberel and liquiritigenin downregulate CXCR4 expression, reducing tumour growth, cell proliferation and invasion. DPN as a selective agonist of the β subunit of the estrogen receptor enhances integrin α and β1, E-cadherin and β-catenin expression, while suppressing the expression of N-cadherin. It leads to intensified adhesion and impaired migration of cancer cells. ER, estrogen receptor; ERBB2, erb-b2 receptor tyrosine kinase 2; ERBB3, erb-b3 receptor tyrosine kinase 3; EGFR, epidermal growth factor receptor; CXCR4, CXC motif chemokine receptor 4; DPN, diarylpropionitrile.

The effects of chemotherapeutics on the expression of membrane proteins are summarized in **Figure 1**.

Anthracyclines can regulate the expression of antigens on the cell membrane surface. Doxorubicin reduces the surface expression of B7-H1 in breast cancer cells to a minimum while upregulating nuclear expression (73). B7-H1 has an anti-apoptotic effect, correlates with tumor invasion and predicts patient survival (74, 75). Therefore, its downregulation and transport of the molecule to the nucleus plays an important role in initiating cancer cell death (73).

Cell death in head and neck squamous cell carcinoma (HNSCC) is mediated by the perforin/granzyme pathway associated with the activity of the membrane pro-apoptotic protein Bcl-2. Tumor resistance correlates with the expression of this protein, which is confirmed experimentally (76). The concentration of the anti-apoptotic protein Bcl-2 was reduced using the combination of cisplatin with 5-fluorouracil (5-FU) indicating the intensification of apoptosis of neoplastic cells (77).

The expression of CEA and MUC-1 increased sharply after treatment with 5-FU (78). CEA is a member of a family of highly related cell surface glycoproteins. It is involved in the adhesion of cancer cells and leads to increased metastasis (79, 80). Upregulation of MUC-2 synthesis is also observed in human colon cancer cells after 5-fluorouracil treatment (81). The use of 5-FU, mitomycin-C or oxaliplatin leads to higher expression of epithelial cell adhesion molecule (EpCAM) and LeY antigen, which is a blood group antigen with a potent expression on the surface of epithelial tumors, including small cell lung cancer (13, 82).

Both betulinic acid (BA) and curcumin inhibit specificity protein (Sp) transcription factors that modulate EGFR expression. The effect reduces the amount of EGFR mRNA produced in bladder cancer cells (83). Downregulation of receptor levels induces cell death through autophagy (84).

#### Nonsteroidal Anti-Inflammatory Drugs

Nonsteroidal anti-inflammatory drugs (NSAIDs) can modulate the expression of membrane molecules. E-cadherin, a transmembrane protein is responsible for the sequestration of β-catenin to the cell membrane and cell adhesion. Its absence leads to the spread of cancer cells as a result of epithelial-mesenchymal transition (EMT) (85). In colon cancer, sulindac protects against the loss of E-cadherin and the accumulation of nuclear β-catenin (86). In contrast, indomethacin and celecoxib reduce E-cadherin expression. This is associated with the invasion and chemoresistance of non–small cell lung cancer (NSCLC) cells following treatment with celecoxib. Results are not optimistic as to the use of celecoxib and indomethacin in lung cancer (87–89).

NSAIDs reduce the expression of the membrane protein EGFR. This results in the inhibition of cell proliferation (90). Sulindac metabolites – sulindac sulfide (SS) and sulindac sulfone (SF) – also downregulate EGFR activation and/or expression in cancer cells, leading to attenuation of EGFR signaling in colon cancer cells (91). Sulindac and celecoxib are involved in the modulation of the physicochemical properties of the cell membrane, which may be associated with anti-cancer properties (89). Licofelone (a novel dual 5-LOX/COX inhibitor) leads to changes in the proportions of saturated, monounsaturated and polyunsaturated fatty acids and increases the cholesterol content of the cell membrane in HCA-7 colon cancer cells. The compound acts *via* the inhibition of EGFR kinase activity, p44-42 MAPK and AKT cascades, which results in a transition to the apoptotic cell-death pathway (92). Indomethacin causes increased E-cadherin expression in colon cancer and up-regulation in pancreatic cancer. It suppresses proliferation and intensifies cell adhesion (93, 94). In summary, regular use of nonsteroidal anti-inflammatory drugs (NSAIDs) is associated with a decreased mortality from colorectal cancer (95).

**Figure 2:**
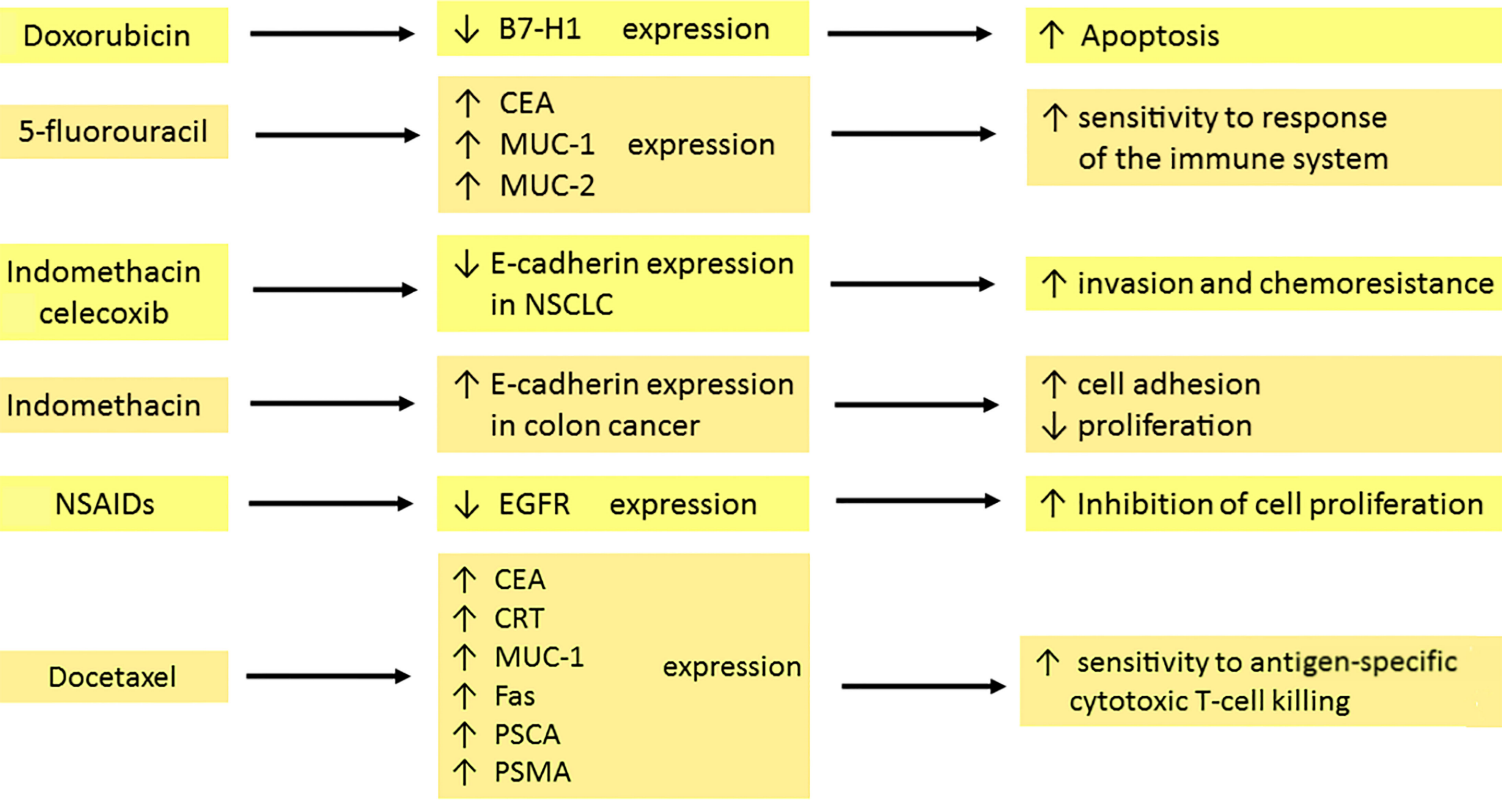
Effects of chemo- and pharmacotherapy of the tumour on the expression of membrane antigens. Doxorubicin, through the inhibition of surface anti-apoptotic B7-H1 expression in breast cancer, is involved in the initiation of cell death. 5–fluorouracil enhances CEA, MUC-1 and MUC-2 expression, which is associated with sensitization of the immune system. Indomethacin and celecoxib decrease the expression of E-cadherin in NSCLC, which leads to amplified invasion and drug resistance development. In contrast, indomethacin in colon cancer increases E-cadherin expression. Therefore, tumour cells have a stronger capacity for adhesion but diminished proliferation. The reduction of EGFR expression by NSAIDs contributes to the inhibition of cell proliferation. Docetaxel upregulates the expression of CEA, CRT, MUC-1, Fas, PSCA and PSMA. The result is a high sensitivity to killing by cytotoxic T cells. carcinoembryonic antigen (CEA), mucin-1 (MUC-1), mucin-2 (MUC-2), non–small cell lung cancer (NSCLC), nonsteroidal anti-inflammatory drugs (NSAIDs), calreticulin (CRT), prostate stem cell antigen (PSCA), prostate-specific membrane antigen (PSMA).

The effects of chemotherapeutics on the expression of membrane proteins are summarized in **Figure 2**.

### Regulation of Multidrug Resistance Proteins by Pharmacotherapy

Multidrug resistance (MDR) is a serious problem that hampers the effective treatment of cancer. It may also increase mortality among cancer patients. MDR is associated with the insensitivity of cancer cells to several groups of drugs. The members of the superfamily of ABC transporters called ATP-binding cassette transporters pump drugs out of the cell. The best-described efflux protein is P-glycoprotein (P-GP) encoded by the gene ABCB1 (MDR1 – multidrug resistance 1). Multidrug resistance-associated protein 1 (MRP1/ABCC1) or ABCG2 also plays a significant role in multidrug resistance (96). Drugs related to ABC transporters include taxanes (e.g., docetaxel and paclitaxel) and vinca alkaloids (vinblastine and vincristine), anthracyclines (daunorubicin and doxorubicin), topoisomerase inhibitors (etoposide and topotecan), and tyrosine kinase inhibitors (dasatinib and gefitinib) (97).

As an integral membrane protein, P-GP is overexpressed on the surface of cancer cells. This is one of the major barriers to achieving a therapeutic drug concentration. Overcoming this barrier would reduce the doses of the chemotherapeutic drug, the number of side effects and shorten the treatment time. Blockers should act selectively on ABC transporters in the tumor. Their efficiency depends on the expression of the transporters. There are several mechanisms of P-GP inhibition. One of them is competitive inhibition that blocks drug binding. Other mechanisms are connected with noncompetitive inhibitors, disturbing the membrane lipid bilayer and downregulating the expression of P-GP (98). Three generations of P-GP inhibitors that inhibit the efflux of drugs have been distinguished. The first generation is not used in cancer therapy but rather in treating high blood pressure, bacterial, fungal and viral infections or allergies (99). Unfortunately, these drugs have to be taken in high doses, which leads to toxicity. The second-generation drugs are more potent and less toxic but interact with the CYP3A4 enzyme and other ABC transporters (100, 101). The third generation of inhibitors is the most specific for P-GP. These compounds have been examined in clinical studies, but with no spectacular results both in reducing toxicity and effectiveness in increasing the overall survival of the patients (102, 103). Investigators are currently researching the fourth generation which involves natural sources/derivatives, peptidomimetics and dual-activity ligands (104, 105).

The limited success of ABC transporter inhibitors in clinical trials is related to the multitude of elements that contribute to multidrug resistance. The use of modulators in clinical trials leads to a slight improvement in the therapy of patients. Importantly, many serious side effects have been reported, making their safety questionable (99). For this reason, the high toxicity of the generation of P-gp inhibitors encourages the study of natural products that can be used as MDR modulators. Recent studies indicate that natural products, such as vegetables, fruits and their components (especially polyphenols or flavonoids), can be used as MDR modulators (106, 107).

Some anti-cancer drugs can regulate the expression of proteins from the MRP family. Under the influence of doxorubicin, the expression of MRP1 transporter increases in small lung cancer. This drug also leads to MRP2 overexpression, which is not detected before treatment (108). In breast cancer resistance protein/ATP-binding cassette sub-family G member 2 (BCRP/ABCG2), expression is upregulated with an increase in the concentration of mitoxantrone used. This correlates with the degree of drug resistance of cancer cells (109). Reduced response to methotrexate treatment is also associated with BCRP protein expression (110). Vincristine resistance results from increased expression of ABCB1 (MDR1), ABCC1 (MRP1), ABCC2 (MRP2) and ABCC3 (MRP3) genes, which may depend on cell type, exposure time, and drug concentration (111).

**Figure 3:**
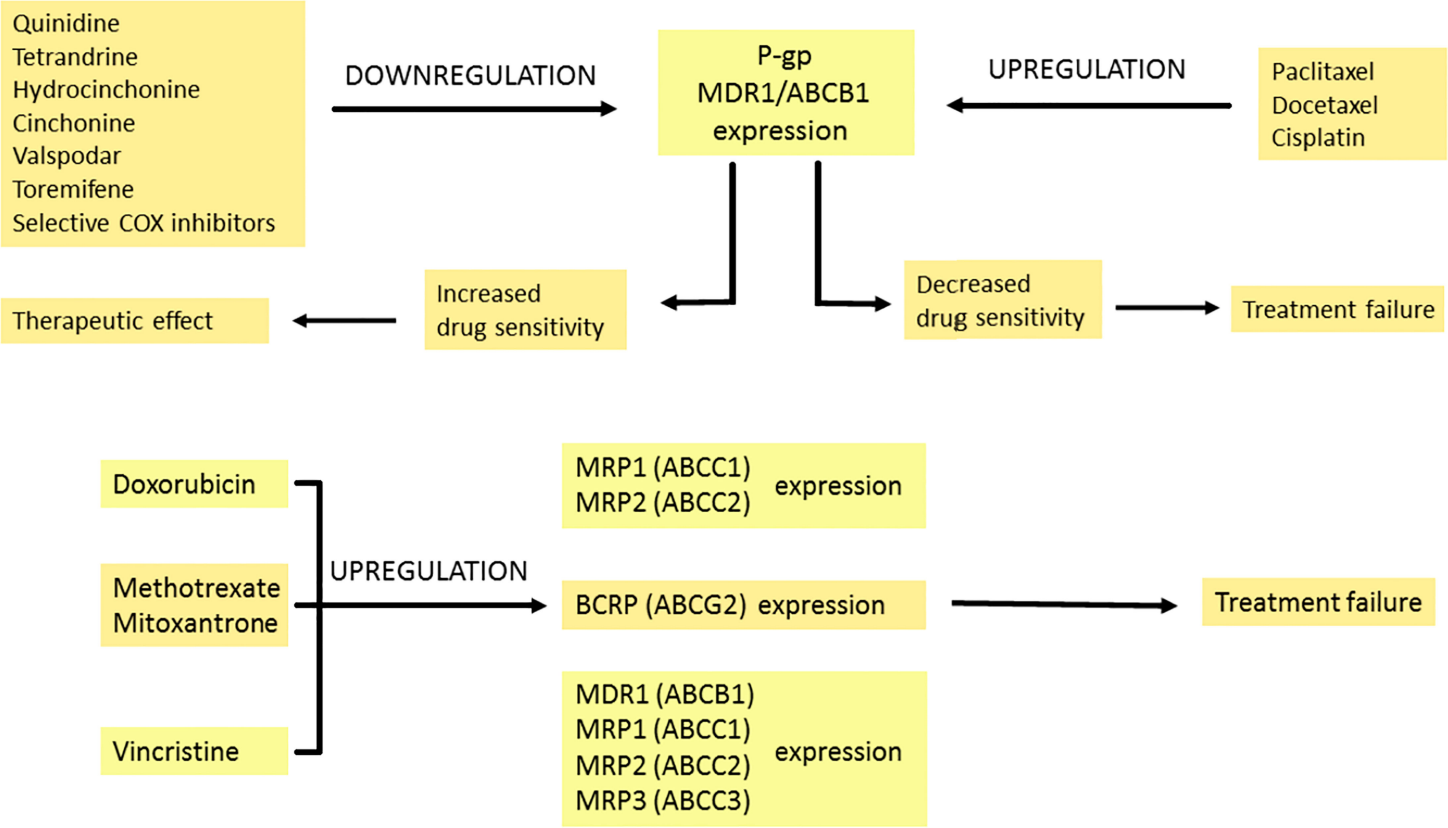
The modulation of multidrug resistance proteins by pharmacotherapy. Expression of P-GP can be increased or decreased by many drugs. Quinidine, tetrandrine, hydrocinchonine, cinchonine, valspodar, toremifene and selective COX inhibitors downregulate P-GP expression, which leads to enhanced transport of the drug into tumour cells and therapeutic effect. In contrast, when the expression of P-GP is downregulated by paclitaxel, docetaxel and cisplatin, it is connected with decreased drug sensitivity and treatment failure. Doxorubicin amplifies MRP1 and MRP2 expression. Methotrexate and mitoxantrone intensify BCRP expression. Vincristine upregulates MDR1, MRP1, MRP2 and MRP3 expression. The modulation effects described above are associated with reduced treatment response. Breast Cancer Resistance Protein (BCRP), P-glycoprotein (P-GP), cyclooxygenase (COX), multidrug resistance 1 (MDR1), multidrug resistance-associated protein 1-3 (MRP1-3).

P-glycoprotein overexpression can be observed as a result of paclitaxel and docetaxel treatment (112). Interestingly, in the cisplatin-resistant ovarian cancer cell line, the expression of P-GP is increased even though the drug is not a substrate of P-GP. Generalized stress response to long-term cisplatin treatment and reactive oxygen species (ROS) production results in P-GP overexpression that induces paclitaxel resistance (113). Lee et al. found that selective cyclooxygenase inhibitors inhibit MDR1 expression and P-glycoprotein in taxane-resistant ovarian cancer, thus sensitizing cells to paclitaxel (114). The effects of chemotherapeutics on the expression of MDR-related proteins are summarized in **Figure 3**.

## Physical Methods and Their Mechanisms

### Treatment With Electric Field

Treatment of the cells with a pulsed electric field (PEF) leads to various biological effects. These include electroporation – both reversible and irreversible (IRE), electrostimulation and changes in membrane properties (115).

Electroporation is the formation of water pores in biological membranes. Depending on cell integrity after PEF treatment, two types can be distinguished: reversible and irreversible electroporation (115). The electric field has to exceed the electroporation threshold to electroporate the membrane. For most human cell lines, 1300 V/cm PEF leads to high-level cell permeabilization (116). In the case of smaller cells, such as human erythrocytes, the EP threshold is much higher (117). *In vitro*, plasmalemma electroporation is analyzed by fluorochrome uptake studies. Depending on the size of the fluorochrome, different pore sizes are analyzed. For instance, Yo-Pro-1 may be used to analyze wider pores than fluo-8 aided calcium ion detection (118). During reversible electroporation, the electropore becomes smaller in time after the end of pulse delivery (119). Therefore, cells are permeable even for a short time after PEF treatment. Flow cytometry makes it possible to analyze the time dependence of the electropore annihilation. On the other hand, irreversible electroporation includes instant induction of necrosis after PEF treatment (115). The reversibility of the process might be analyzed by the long-term assessment of cell permeability. For instance, a previous work by the present authors showed the potency of trypan blue kinetic staining studies in the differentiation between reversible and irreversible electroporation protocols (115).

Treatment with electric field may be used to modulate membrane antigens in two distinct attempts. The first includes the electroporation of the cells with the plasmid encoding a specific molecule (for instance, a cytokine such as IL-12), followed by cytokine expression and release (120–122). In this case, electroporation does not directly affect the membrane, but *via* the stimulation with an exogenous molecule, it modulates the systemic response to cancer antigens and sensitivity of the immune cells to the paracrine signal (123). Similarly, Pen et al. showed that electroporation with mRNA encoding constitutively active TLR4 may be used to activate dendritic cells (124). Besides, the immune response of the cell might also be modulated by electroporation with siRNA. Dannull et al. showed that it is possible to modify the antigen processing system in dendritic cells by electroporation with immunoproteasome-targeting siRNA (125). DNA encoding a variety of other molecules may also be electroporated to induce specific modifications to the immune response. For instance, when Tanning et al. electroporated HIV-1 antigen with the ectodomain of PD-L1, anti-HIV-1 response was enhanced in comparison to control samples (126). There is an entirely new concept in which electroporation supports the action of DNA vaccines *via* an additional stimulation of the immune system (127). All the abovementioned effects arise from the changes in the composition of membrane antigens.

Interestingly, aside from the cellular effects of PEF treatment, irreversible electroporation is involved in the increased concentration of cancer-specific molecules in the tumor microenvironment. This allows the immune cells infiltrating the tumor to process cancer molecules and extensively work as antigen-presenting cells, therefore increasing the immune recognition of the tumor. The method is currently widely examined due to its simplicity and possible use as an adjuvant cancer therapy. The mechanism of IRE is slightly different from reversible electroporation. Here, the release of cancer-specific molecules to the tumor microenvironment induces the immune response against cancer. In this case, the immunophenotype of immune cells changes to one set to kill the alien cells (128). Hester et al. proved that IRE may transiently alleviate the immune suppression and enables the activation of T-cells in the tumor site (129). Curiously enough, Zihao Dai et al. proved that IRE of post-ablation hepatocellular carcinoma induces CD8+ cell response against cancer *via* the release of danger-associated molecular patterns (DAMPs) and the downregulation of Treg and PD-1+ cells in the tumor tissue (130). However, the release of damage-associated molecular patterns to the extracellular compartment activates cytotoxic T-cells, thus modifying their cell membrane antigens toward the cytotoxic phenotype of the membrane. There are several types of DAMPs, including histones, genomic DNA, HMGB1, IL1a, IL33, ATP, F-actin, Cyclophilin A, HSP, uric acid, mitochondrial DNA and calreticulin (131). Molecules originate from different compartments of the cell, like nucleus, cytosol or mitochondria (132). These flow out from the cells after cell damage to the extracellular space. Afterward, mentioned DAMPs are recognized mostly by Toll-like receptors (TLR) as well as RAGE, TIM3, P2Y2, P2X7, DNGF1, CD147, CD91, SERC1, FEEL1 and NLRP3 molecules (131). Interestingly, Yimingjiang et al. claim that the effects of nsPEF are similar to PD-1 blockade in the treatment of hepatocellular carcinoma (133).

When EP is combined with the administration of chemotherapeutics, the process is called electrochemotherapy (ECT) (134). The therapeutic agent might be administered intravenously or locally to the tumor site. Some of the most commonly used chemotherapeutics include bleomycin for melanoma treatment or calcium chloride for clinical trials in pancreatic cancer management (135, 136). The effects of ECT on the modulation of membrane antigens in the anticancer response are similar to those achieved in standalone electrochemotherapy. Gerlini et al. proved the activation of dendritic cells following electrochemotherapy of melanoma (137). However, the difference between ECT and EP in terms of modulation of membrane antigens is the higher cytotoxicity induced by ECT related to the administration of the chemotherapeutic agent (118). The cytotoxicity may also affect the composition of membrane antigens.

Intracellular effects of PEF treatment include the permeabilization of intracellular organelles, stimulation of the biosynthesis of specific proteins and modulation of antigens. The first effect was widely described in the Jurkat cell line, in which the nanosecond pulses induced the permeabilization of the mitochondrial outer membrane and, therefore, the release of cytochrome c and reactive oxygen species to the cytoplasm (138). After the disruption of cell membrane integrity, the cell increases the biosynthesis of the components of its cytoskeleton (139). However, in short-term response, Kiełbik et al. proved that reorganization of actin fibers occurs after electroporation (118). The effect might be a response of the cell to unfavorable conditions. Besides, nanosecond pulsed electric field may also affect the formation of enhanced speckle (140). A similar effect relates to the permeabilization of the endoplasmic reticulum. Conversely, protein biosynthesis requires high amounts of energy, thus the PEF stress-induced inhibition of translation serves as a mechanism for cell survival (141). Moreover, several biological effects also arise from the application of electric fields, for instance, eIF2α phosphorylation and 4E-BP1 dephosphorylation (142). Moreover, other studies suggest that nsPEFs activate the major MAPK kinase downstream signaling pathways *via* p38, JNK and ERK (143, 144). From the mechanistic point of view, however, the mechanisms through which nsPEF modulates the activity of membrane kinases remain unknown. Some authors hypothesize that the effect occurs *via* the induction of cell stress and thus the activation of kinases. Also, studies by Vernier et al. prove that the biological changes may arise from the increased concentration of cytoplasmic calcium (145). A similar tendency was observed by Morotomi-Yano et al. who proved the activation of AMPK with the increased content of cytoplasmic Ca^2+^ (146). The study introduces an entirely new mechanism in which the stimulation with nsPEFs may modulate the immunophenotype of the cells. AMPK is one of the critical regulators of vesicle trafficking, which is why its inhibition disallows the proper membrane-vesicle interaction and thus in the long term affects the composition of membrane antigens (147). Moreover, nsPEFs may regulate the phenotype of the membrane and presumably the content of antigens in chondrocytes *via* the Wnt/β-catenin pathway (148). Interestingly, an example of direct modulation of membrane antigen by nsPEF is the CD95 death receptor which could be modulated in Jurkat and U937 cell lines (149). Significant effects and high expectations based on the aforementioned studies prove that much research has yet to be done in this field.

nsPEF also exerts an effect on protein antigens and changes membrane lipid composition *via* the interaction with scramblase (150), an enzyme responsible for the transport of negatively charged phospholipids between the inner and outer leaflet of the membrane. As a consequence, the lipid profile of the plasmalemma alters.

**Figure 4:**
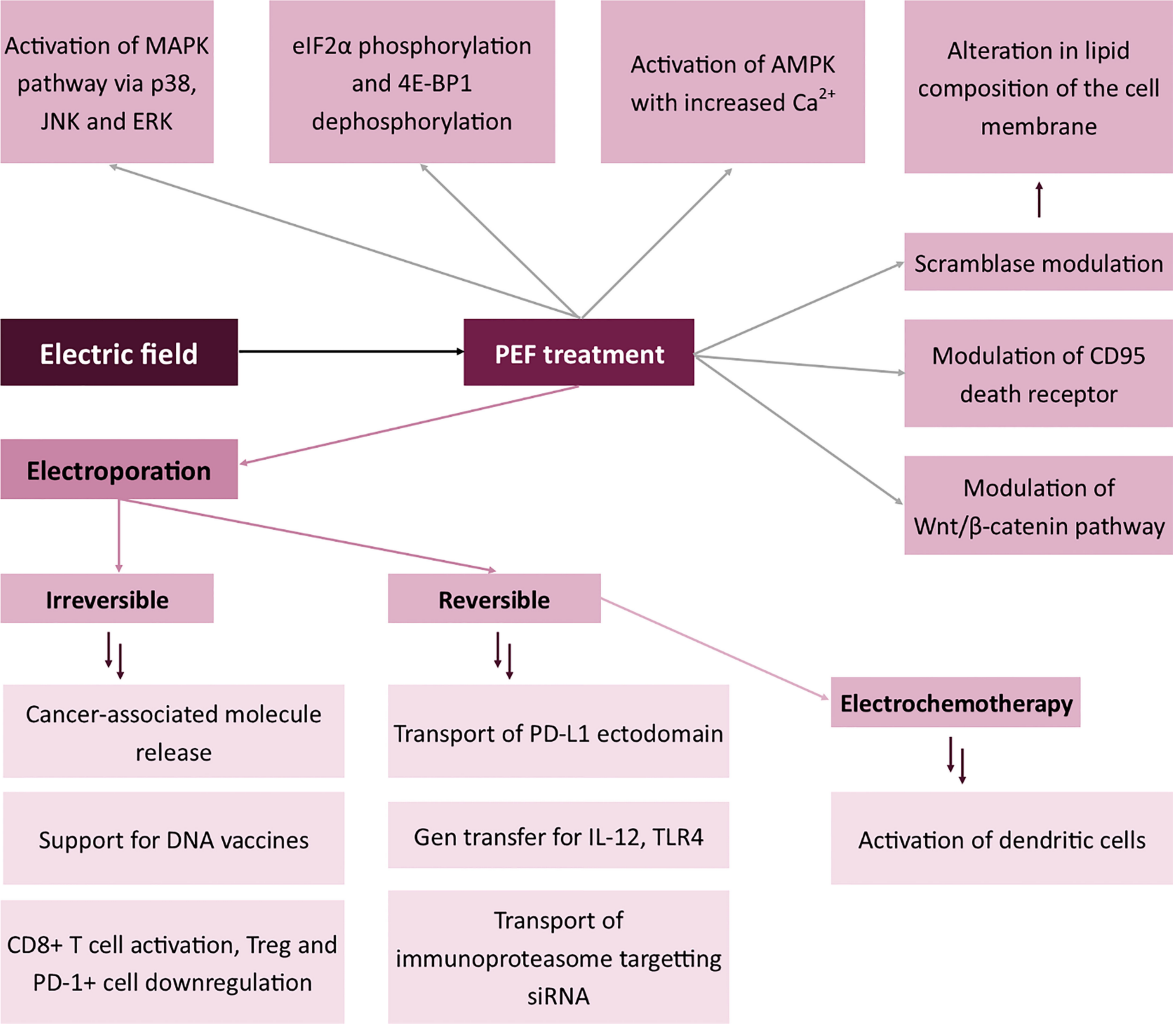
Effects of electric field on the expression of membrane antigens. Mitogen-activated protein kinase (MAPK), c-Jun N-terminal kinase (JNK), Extracellular signal-regulated kinase (ERK), Eukaryotic Translation Initiation Factor 2A (eIF2α), Eukaryotic translation initiation factor 4E-binding protein 1 (4E-BP1), 5’ AMP-activated protein kinase (AMPK), Programmed death-ligand 1 (PD-L1), Toll-like receptor 4 (TLR-4).

All the mentioned mechanisms in which the electric field modulates the immunophenotype of the cells are summarized in **Figure 4**.

### Photodynamic Reaction

Photodynamic reaction (PDT/PDR) includes the irradiation of the photosensitizer with an adequate wavelength light. The physical mechanism of PDR includes the shift of electrons to the high-energy state forwarded by the emission of excess energy to biomolecules (151). The process is responsible for the irreversible oxidation of intracellular components and even redox changes in the DNA. Without proper genome editing and cell repair system, cancer cells die from the accumulation of invalid proteins and genetic material (152). In the case of the cellular changes induced by the photodynamic reaction, various membrane proteins undergo alternations. For instance, actin bound to the membrane by zyxin was proved to change its structure dramatically after the irradiation of melanoma cells with curcumin (153, 154). Moreover, PDT/PDR induces the stress response of the irradiated cells and therefore changes in the cytoskeleton which prevents the cells from physical disruption of their integrity (153).

In PDT, an inactive anticancer agent is administered systemically or locally. Even in systemic administration, PDT is a highly selective treatment as the local light irradiation goes in parallel with the accumulation of photosensitizer within a tumor. Still, if the local administration of the photosensitizer to the tumor site is possible, it decreases the systemic side effects (151).

PDT influences the immunostimulatory attributes of antigen-presenting cells, thus alleviating autoimmune diseases (155). Mroz et al. described the effects of PDT on cancer cells and the immune system *in vivo* (156). The authors demonstrated that PDT induces the increased killing of cancer and the production of TNFα and IFNγ in the tumor tissue. Moreover, PDT elicited the development of epitope-specific CD8+ cells followed by the destruction of distant untreated antigen-positive tumors. The much lower CTLs-related effects on antigen-negative cells prove that PDT induces changes in immune cell membranes thus allowing for the higher cytotoxicity toward the tumor. High selectivity and specificity of PDT toward the antigen was demonstrated also by other authors, for example, Kabingu et al. The group showed that PDT enhances the systemic recognition of Hip1, a tumor antigen associated with basal cell carcinoma (157). In another study, Mroz showed that PDT induces a potent epitope-specific response against testis antigen P1A and simultaneously toward cancer (158). Reginato et al. presented a method of combining Treg depletion by cyclophosphamide with PDT to potentiate PDT-mediated immunity (159). Wang et al. also presented an interesting attempt to enhance the immunomodulatory effects of PDT. The authors engineered an antigen-photosensitizer nanocarrier to facilitate ROS-triggered immune response to PDT (160). Remarkably, the antitumor effect of antigen-specific immunization may be enhanced by the use of antigen-specific conjugates – as demonstrated by Wang et al. in their successful attempts of targeting PSMA on prostate cancer cells (161).

Concerning the mechanism of PDT-aided immunity and therefore the activation of immune cell cytotoxicity (also membrane cytotoxicity-related antigen expression), Korbelik suggested that the vigorous innate immune reaction arises from the intensified phagocytosis of dead tumor cells (162). Zhang et al. provided evidence that PDT also induces presentation of surface MHC I related antigens and enhances antigen processing through the restoration of TAP1 protein expression in glioma cells (163). TAP1 can potentiate the transport of new antigen peptides and therefore is involved in PDT-related immunomodulation (163). The generation of ROS plays a significant role in PDT-induced immunity (160). Interestingly, some photosensitizers, such as protoporphyrin IX, may be selectively accumulated only in activated lymphocytes. The same authors proved the functional alternations in antigen-specific and nonspecific immune components (164).

Aside from cancer-related changes in the cell membrane, PDT may also be used to induce the immunosuppression of contact hypersensitivity (165). Light radiation was proved to induce genomic changes, leading to the induction of POMC expression (166). When keratinocytes are irradiated with UVB light, POMC is extensively biosynthesized *via* a p53-related pathway and converted to αMSH or ACTH afterward (167, 168). Therefore, aside from the local effects, the light might induce systemic effects – such as the increased biosynthesis of corticosteroids (169).

**Figure 5:**
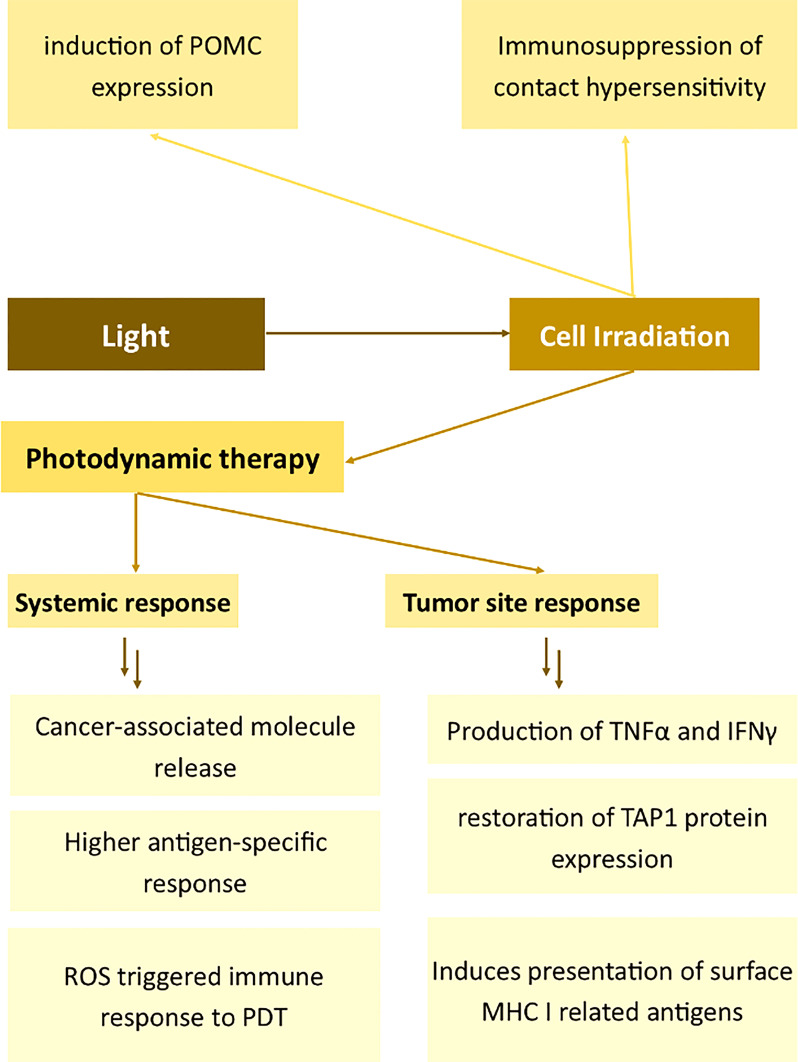
Effects of light on the expression of membrane antigens. Proopiomelanocortin (POMC), Tumour Necrosis Factor α (TNFα), Interferon γ (IFNγ), Transporter associated with antigen processing 1 (TAP1), Major Histocompatibility Complex I (MHC I), Photodynamic therapy (PDT).

The mechanisms in which light affects the immunophenotype of the cells are summarized in **Figure 5**.

### Radiation Therapy

Radiation therapy involves the application of high-energy waves (like X-ray or γ-waves) or particles (like electrons, α particles, β particles or protons) against cancer. Early clinical attempts showed the advantages of X-rays and alpha particles over the beta particles, thus more research studies concerned the more effective techniques.

#### X-Ray Radiation

X-ray radiation rises from the emission from the atomic nuclei. Roentgen radiation involves the emission of X-ray waves (wavelengths ranging from 0.01 to 10 nm) and the administration of the radiation dose to the tissue (170, 171). There is no evidence that X-ray induces the poration of the membrane – instead, it exerts effects on the genetic material of the cell and the redox potential in the cytoplasm (172, 173).

In non-cancerous cells, the genetic material repair system is valid and radiation effects are reversible (174–176). The only side effects of X-ray treatment include skin burns and the induction of neoplasms in susceptible patients (177, 178). Conversely, in cancer cells, DNA-repair systems are often impaired, therefore X-ray treatment induces irreversible damage to the genetic material (179, 180). Neoplasms develop and the abnormal cells accumulate in the irradiated site. The changes in the genetic material induce the biosynthesis of redox-related proteins as well as free radical scavengers (181). The composition of surface antigens is also changing. Irreparable changes occur in the cell, and the neoplasm induces the expression of death-related molecules (182).

Studies on a murine model demonstrated that the immune response following irradiation relates to the locus of the antigen affinity (183). Namely, antigens associated with the H-2 locus were much more resistant to irradiation than those related to H-1 and H-3 loci. The author shows the modulatory role of X-ray on membrane antigens in relation to transplantation immunity (183). Other papers also prove decreased rejection rates (ratio of unsuccessful transplantations) in patients after Total Body Irradiation (TBI) (184). The mechanism of the effect of X-rays on cell immunophenotype may also be related to changes in proliferating cell nuclear antigen (PCNA) and thus the proliferation phenotype in V79 hamster fibroblasts (185). Also, Miura et al. showed that PCNA is involved in the repair mechanisms after X-ray radiation and thus in the proliferation properties of human fibroblasts (186). Since cells change membrane antigens as they proliferate, all the mentioned processes have to relate to changes in the composition of membrane antigens. On the other hand, Shreder et al. described the lack of the effects of X-rays on transcription factors such as PPARγ, C/EBPα and C/EBPβ in human pre-adipocytes (187). However, the authors claim the modulation of cells by inflammatory mediators rather than genomic factors (187). Interestingly, studies on osteoblasts demonstrated that X-ray irradiation induces the differentiation of cells *via* the RhoA pathway and the change in the cytoskeleton (188).

X-ray radiation also had a direct effect on cancer-related molecules on the cell membrane of human gastric cancer cells causing an increase in CEA membrane content and elevating the level of MHC I (189). Wittenborn et al. showed an autoimmune phenotype achieved in mixed chimaera models after ionizing X-ray radiation and Cesium-137 treatment (190). Finally, Tandl et al. show that X-ray elicited calcium ion signaling cascade and, in consequence, activated human T-lymphocytes (191).

**Figure 6:**
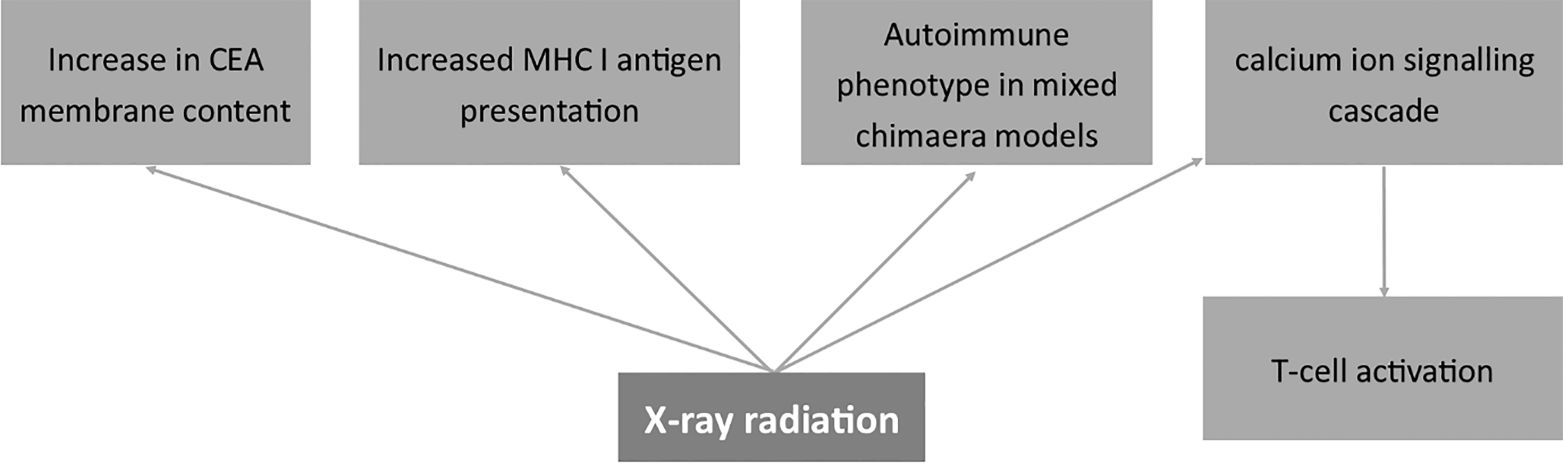
Effects of X-ray radiation on the expression of membrane antigens. Carcinoembryonic antigen (CEA), Major Histocompatibility Complex I (MHC I).

The mechanisms in which X-rays modulate membrane antigens of cells are summarized in **Figure 6**.

#### Gamma Radiation

Gamma radiation may be generated with isotope-based generators, like Cobalt 60 (192). γ radiation exerts dualistic effects on human cells, depending on the dose of the radiation and the expression of REG4, BIRC5 and NEIL2 genes (193). Long term low doses of γ waves exposure induces carcinogenesis in the healthy organism. Daniels et al. showed that the protracted exposure to low-dose gamma radiation is significantly associated with leukemia (194). Conversely, annual outdoor ambient gamma dose rate is not associated with childhood leukemia in German population nor increased the risk in United Kingdom children (195, 196). However, when the dose significantly increases and is administered to the tumor site, it induces apoptosis in cancer cells and is being used in the anticancer therapy. Interestingly, both effects are observed during the therapy and not only cancer is affected by the radiation, but the non-cancerous cells as well (192). Cancer cells gain susceptibility to the γ radiation therapy, when it is combined with other modalities of treatment. For instance, studies by Hofmanova et al. proved the increased susceptibility to γ radiation when the cells were simultaneously treated with lipoxygenase inhibitors (197). Besides, curcumin enhances the cytotoxic effect of radiation in MCF-7 cells (198). According to the molecular changes induced by gamma radiation, CuZnSOD and MnSOD proteins expression increases in the time-dependent manner (199).

#### Nuclear Medicine

Like X-ray radiation, nuclear medicine involves the irradiation of cancer cells, however, in this case with α particles instead of X-ray waves (200, 201). In the past, β radiation was used for studies on therapeutic applications (200). In nuclear medicine therapy, a radioactive isotope emits radiation, driving cancer cells to apoptosis and causing damage to the genetic material of the tumor.

Depending on the stage of tumor differentiation, the isotope may be administered locally or systemically. In well-differentiated cancers, such as hyperthyroidism and differentiated thyroid cancer, the most commonly applied isotope – I-131 – is captured nearly fully in the thyroid cells (202). Radioisotope might be conjugated with a molecule specifically captured by the organ. For instance, adrenal cortex cells uptake 131I-6-β-iodomethyl-19-norcholesterol or 6-methyl-75Se-methyl-19-norcholesterol which is used in the diagnostic scintigraphy of the adrenal cortex (203). The method allows for the synthesis of more specific therapeutics and decreases the number of systemic side effects of the therapy. Moreover, highly effective iodine conjugates, such as I-Metaiodobenzylguanidine (131I-MIBG) are applied in targeted radiotherapy for children with neuroblastoma (204). Aside from tumors, radioiodine might be used for the therapy of Graves’ Disease (205).

### Hyperthermia and Hypothermia

Hyperthermia is a state of the body where the temperature is elevated to induce a specific response. In pathology, it occurs systemically in fever (206–208). However, studies by Muckle et al. prove that hyperthermia may also be artificially induced as part of anticancer therapy (209). Several methods may be used to elevate the body’s temperature – electric field, light radiation and heat convection. When the temperature remains exceptionally high, the cancer tissue undergoes necrotic death and immunization is similar to that obtained in IRE or other ablation techniques (210–214). The parameters of the applied heat suppliers have to be carefully set (215). Depending on the applied temperature, heating methods may be divided into lethal and sub-lethal hyperthermia. Both processes drastically differ in biological response of the cancer cells. Sublethal temperatures induce the epithelial to mesenchymal transition in breast cancer cells, simultaneously increasing the chemosensitivity of the cells (216). Henle et al. proved that sub-lethal radiation or heat damage may become lethal when treated with 40oC after the damage or may be repaired when treated with lower temperatures (217). The tendency that sub-lethal hyperthermia increases the cytotoxic effect of radiation damage was also proved by the other authors (218). The control of quiescent cells by sublethal hyperthermia is useful for suppressing the repair of both potentially lethal and sublethal damage (219). Like in IRE, lethal hyperthermia-aided ablation induces systemic response to tumor antigens and may be potentially used in the treatment of metastatic disease (220). The effects of hyperthermia may be enhanced by dendritic cell immunotherapy following hyperthermia (221). Moreover, the release of DAMPs to the extracellular space induces an enhanced cytotoxic phenotype of immune cells and an increased infiltration of the tumor microenvironment (222). Moreover, photothermal ablation modulates the intratumoral myeloid population toward tumor immunogenicity (223). On the other hand, multiple exposures to radiofrequency radiation hyperthermia suppressed cell-mediated immunocompetence (224). The immune system response is not evoked exclusively by extremely high temperatures. Also, studies by Umar et al. proved that febrile temperature induces CD4 T-cell differentiation regulated by the TRPV channel and the Notch signaling pathway (225). Hyperthermia also increases the presentation of HLA-DR in NK and NKT cells (226). The mechanism of action of hyperthermia also relies on Heat Shock Proteins (HSP) and thus indirectly on MHC I (227). Extracellular HSP70 serves as a link between NK and dendritic cells *via* the induction of NKG2D ligand and MHC class I chain-related protein A overexpression and the augmentation of IFN-γ release (228).

On the other hand, hypothermia is known to inhibit the classical complement pathway. Besides, it is associated with decreased expression of pro- and anti-inflammatory effectors (229).

**Figure 7:**
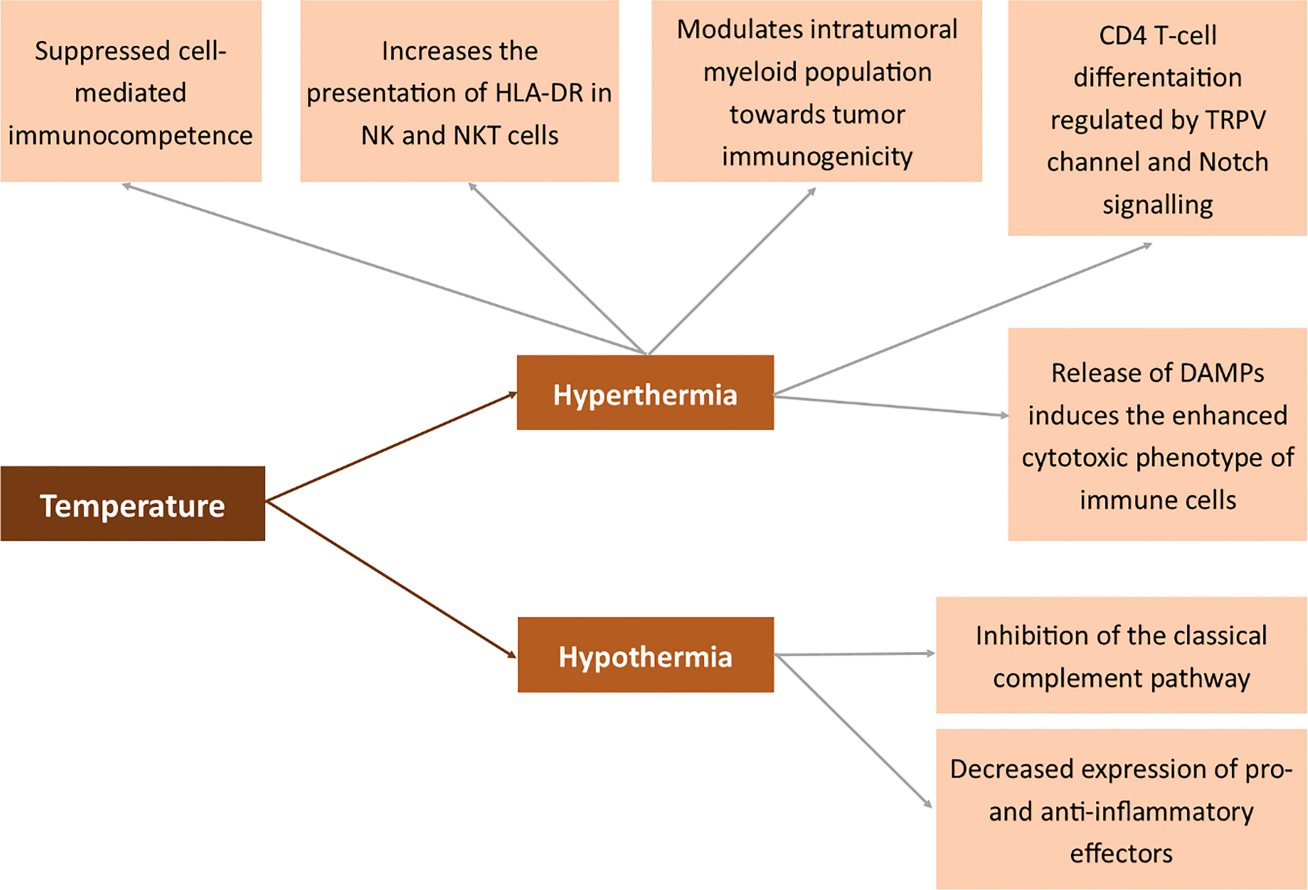
Effects of temperature on the expression of membrane antigens. Human leukocyte antigen DR (HLA-DR), transient receptor potential cation channel (TRPV), Damage-associated molecular pattern (DAMP).

All the abovementioned mechanisms in which temperature affects the membrane antigens are summarized in **Figure 7**.

### Magnetic Field

Treatment of cells with magnetic field (MF) is not fully covered in the literature yet. There are three types of MF – alternating (AMF), pulsating (PMF) or static magnetic field (SMF). In the case of PMF, the effects are nearly identical to the ones in the electroporation technique (230). On the other hand, AMF and static MF have not been analyzed in anticancer therapy yet. Only a few papers show the potential of magnetic field in the induction of cell death (231, 232).

Several authors combined MF treatment with other anticancer modalities, such as electroporation, and obtained favorable results in permeabilizing cells (233). Besides, specific MF-dependent nanovesicles are synthesized to enhance the anticancer effect of the therapy severely targeting melanoma (234). Aside from the effects on ferromagnetic metal-containing enzymes, MF also induces local hyperthermia and results in the ablation of the tissue (231). All the cytotoxic effects relate to the change of membrane antigen composition to the one connected with cell death.

### Ultrasound Treatment

Sonodynamic reaction (SDR) takes place when ultrasounds act on the cells in a medium containing ultrasound-activated compounds – sonosensitizers (235, 236). The effects of ultrasounds on human tissues are much more complex than in PDR. Ultrasounds act both on the sonosensitizer (in SDR) and systemically (237, 238). When the sono-active compound gets to the cell, ultrasound treatment induces implosion and sonoluminescent light emission (239–241). Moreover, the collection of diffused gases leads to forming a high-volume gas bubble in a process called cavitation (242).

Aside from specific antigen response, the cell prevents itself from future disruption, and therefore the increased biosynthesis and organization of the cytoskeleton takes place. Mechanisms in which ultrasounds modulate membrane antigens may relate to a ROS burst after SDR or the activation of autophagy (243, 244). Li et al. proved that autophagy in the case of SDR may relate to the translocation of TFEB induced by a ROS burst following SDR (245). Also, hypoxia may relate to the modulatory effects of SDR (246). Several attempts were made to stimulate immunity after SDR. One of them was the generation of a two-dimensional coordination nanosheet which additionally activated the immune response *via* the utilization of the TLR9 agonist in its structure (247).

Boiling histotripsy of the tumor tissue *via* the application of ultrasounds is similar to the effects of IRE (248). The mechanism is similar and the methods seem complimentary and both modulate the immunophenotype of CD8+ cells to a phenotype which is more cytotoxic toward cancer cells. Also, high-intensity focused ultrasound therapy seems to modulate the activation of CD8+ and CD4+ cells infiltrating the tumor tissue (249). Moreover, HIFU induced the increased expression of Nlrp3, Jun, Mefv, Il6 and Il1β and alterations in macrophage polarization in studies by Fite et al. The authors also described the upregulation of several immune pathways involving Nod1, Nlrp3, Aim2, Ctsb, Tlr1/2/4/7/8/9, Oas2, and RhoA (250). Studies by Ji et al. showed a shift from polarized M2 macrophages into M1 phenotype and depletion of myeloid-derived suppressors in the tumor microenvironment following the application of ultrasounds (251). In the case of immune cells, nitric oxide (NO) is responsible for the shift in the functioning of immune cells (251).

At the other end of the spectrum of ultrasound use is sonoporation (252). The process involves the formation of a pore in the cell membrane with the use of ultrasounds. Mechanisms involved in sonoporation include cavitation, shear stress to the membrane, endocytosis and finally mechanical stress to the membrane (253). The method seems very promising; however, due to its novelty, only several studies concern the application of ultrasounds in the modulation of membrane antigens. The first attempt includes the transport of antigen mRNA to dendritic cells, which afterward, *via* the expression of antigenic peptide, can act as DC vaccines against tumor cells *in vivo* (254). Research also concerned the transport of siRNA molecules to the primary T cells which resulted in a decreased methylation-controlled J protein expression (255). The Golgi-associated transmembrane protein downregulation is involved in increased resistance to specific drugs by inducing expression of the ABCB1 drug transporter *via* the c-Jun-related pathway. Therefore, sonoporation may be used to modulate drug-resistant proteins (256).

**Figure 8:**
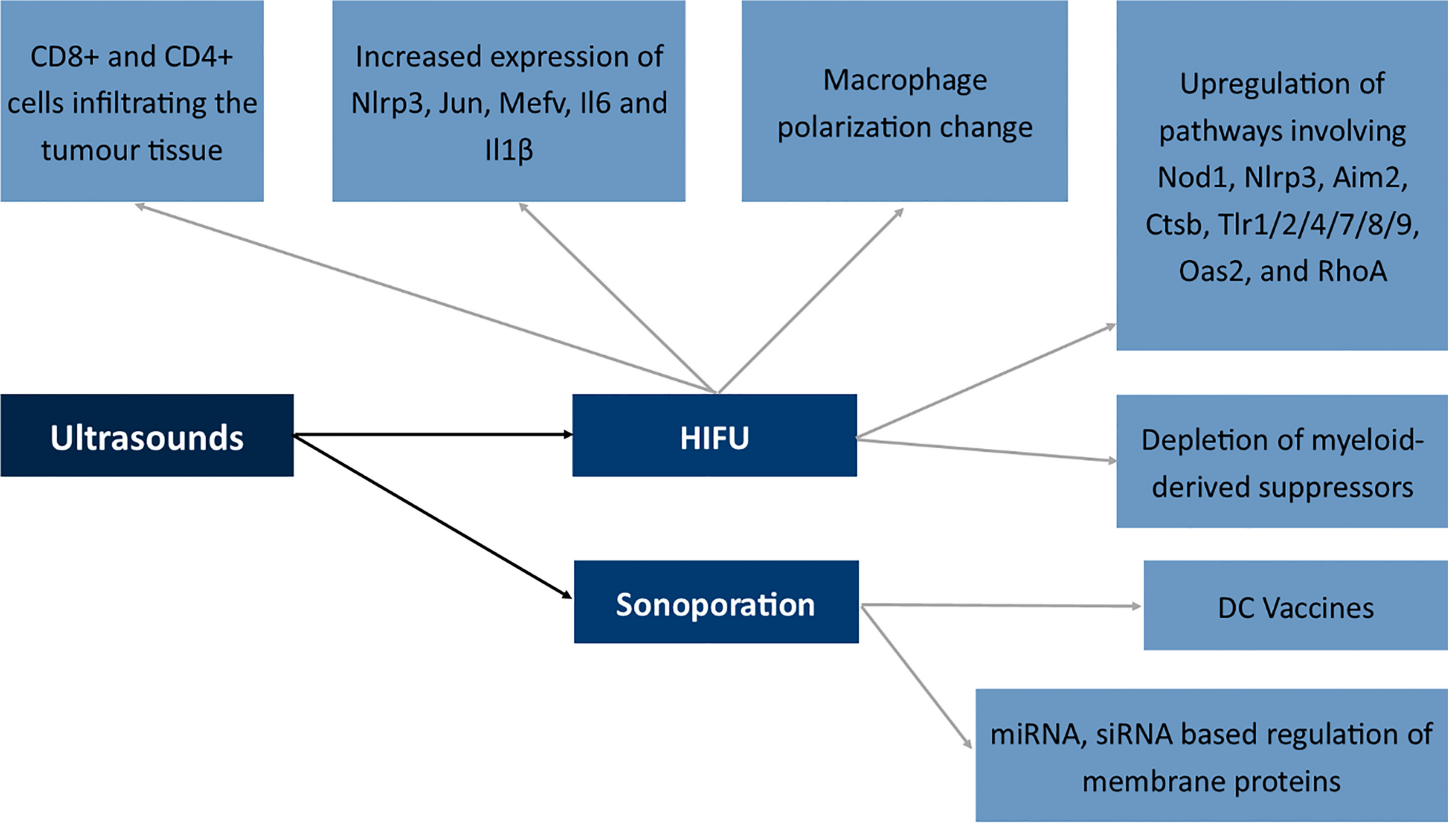
Effects of ultrasounds on the expression of membrane antigens. NLR Family Pyrin Domain Containing 3 (Nlrp3), Jun Proto-Oncogene (Jun), Mediterranean fever gene for marenostrin (MEFV), Dendritic cell (DC), Nucleotide-binding oligomerization domain-containing protein 1 (Nod1), Absent In Melanoma 2 (Aim2), Cathepsin B (Ctsb), Toll-like receptor 1/2/4/7/8/9 (Tlr1/2/4/7/8/9), 2’-5’-Oligoadenylate Synthetase 2 (Oas2), Ras homolog family member A (RhoA), High-intensity focused ultrasound (HIFU).

All the mentioned mechanisms in which ultrasounds modulate the membrane antigens of the cells are summarized in **Figure 8**.

### Modulation of Membrane Antigen in Clinical Practice

Modulation of membrane antigens has found a variety of clinical applications. Affected membrane proteins and receptors might be used in the production of lymphocytes used in cell-based therapies or sensitization of the tumor toward chemotherapy. In general, the modulatory effect is, in most cases, the side effect of the used therapy. Sometimes the result is profitable – for instance after PDT or PEF treatment and in some cases, the effect is negative and leads to the increase in aggressiveness of cancer – like sometimes after hormonal therapy.

CAR-T or CAR-NK cells therapies target cancer-specific antigens with the use of T- or NK-cells with a newly introduced CAR receptor. In the process of CAR design, the new receptor has to be specific to the targeted cells and not self-destroy the CAR-T/NK cells. The therapeutic cells easily find their application in leukaemia or in the treatment of other haematological malignancies. For instance, in B-ALL, cytotoxic cells often target CD20 or CD19 antigens (12). Most T-ALL CAR target the CD7 molecule which is also present on CAR-T cells (257). Therefore, to avoid fratricide during the production of anti-CD7 CAR-T cells, self-reactive CD7 molecules are depleted by gene silencing methods or deleted with the CRISPR-Cas system. A similar approach may be implemented in the production of anti-CD5 or anti-CD2 CAR-T cells. Cell-based immunotherapy presents several limitations in terms of antigen expression. First, the efficacy of immunotherapy depends on the number of targeted receptors. Second, the therapy has to be personalized – the cells should be compatible with the HLA system of the patient, and thus most of CAR-T cells are produced from patients’ own T-cells. The third problem is the low efficacy of targeting solid tumors. There are a few factors that contribute to it – a low abundance of solid-tumor specific antigens and low infiltration of CAR-T cells to the tumor site. To overcome the problems, researchers investigated the methods for increasing cancer-specific molecules and their immunogenicity. The attempt was applied in clinical practice, and several clinical trials involved the modulation of membrane antigens *via* the application of pharmacotherapy and biological therapeutics. **Table 1** summarizes completed clinical trials in the modulation of membrane antigens.

**Table 1:** Summary of antigen loss or modulation found in published clinical trials.

Number of Patients	Therapy Protocol	Target Antigen	Short Description	Ref.
10	Biological: INO-3112 Device: CELLECTRA™-5P	CD 107a, granzyme B and perforin	Immunogenicity of INO-3112 DNA vaccine delivered by electroporation to participants with HPV associated with HNSCC	Bagarazzi et al. (258), NCT02163057
170	Drug: gp100:209-217 (210M) Drug: Montanide ISA-51 Drug: IL-2 Drug: MART-1:26-35(27L) Biological: Abl cells Drug: Fludarabine Drug: Cyclophosphamide Biological: GCSF (Growth colony-stimulating factor) Procedure: Apheresis	FoxP3, CD25 and CTLA-4	The impact of CD4(+)FoxP3(+) regulatory T cells on human antitumor immune responses	Yao et al. (259), NCT00001832,
53	Biological: Anti-Cluster of Differentiation (CD)19-Chimeric antigen receptor (CAR)	CD19	CD19-CAR T-Cell Therapy in Children and Young Adults With B-ALL	Kowolik et al. (260), NCT01593696
8	Drug: indium-111-ibritumomab tiuxetan Drug: 90Y Zevalin	CD20	Modulation of CD20 Expression in Plasma Cells of Patients with Multiple Myeloma	Treon et al. (261), NCT01207765
112	Biological: CART-TnMUC1 Drug: Cyclophosphamide Drug: Fludarabine	TnMUC1	CART-TnMUC1 in Patients with TnMUC1-Positive Advanced Cancers	Gutierrez et al. (262), NCT04025216
30	Biological: GPC3 and/or TGFβ targeting CAR-T cells	GPC3	GPC3-CAR-T Cells for Immunotherapy of Cancer with GPC3 Expression	Pang et al. (263), NCT03198546
1260	Biological: Pembrolizumab	PD-1	Pembrolizumab (MK-3475) in Patients with Progressive Locally Advanced or Metastatic Carcinoma, Melanoma, or Non-small Cell Lung Carcinoma	Patnaik et al. (264), NCT01295827
30	Biological: Anti-B-cell maturation antigen (BCMA) chimeric antigen receptor (CAR) T cells	BCMA	T Cells Targeting B-Cell Maturation Antigen for Previously Treated Multiple Myeloma	Abbas Ali et al. (265), NCT02215967
73	Biological: CART-19	CD19	Chimeric antigen receptor T cells for Patients with CD19+ Leukemia and Lymphoma	Shannon et al. (266), NCT01626495
26	Biological: CART-19	CD19	CART19 to Treat B-Cell Leukemia or Lymphoma	Frey et al. (267), NCT01029366
208	Biological: CD22-CAR	CD22	Anti-CD22 Chimeric Receptor T Cells in Pediatric and Young Adults with CD22-expressing B Cell Malignancies	Fry et al. (268), NCT02315612
93	Biological: gene-modified T cells targeted	CD19	CD19 CAR Therapy in Acute Lymphoblastic Leukemia	Park et al. (269), NCT01044069
21	Biological: Anti-MAGE-A3-DP4 TCR PBL Drug: Cyclophosphamide Drug: Fludarabine Drug: Aldesleukin	MAGE-A3	T Cell receptor immunotherapy targeting MAGE-A3 for patients with metastatic cancer	Yong et al. (270), NCT02111850
11	Biological: ETBX-051; adenoviral brachyury vaccine Biological: ETBX-061; adenoviral Mucin-1 (MUC1) vaccine Biological: ETBX-011; adenoviral Carcinoembryonic antigen (CEA) vaccine	CEA, MUC1, Brachyury	Multitargeted recombinant Adenovirus 5 (CEA/MUC1/Brachyury) ‐based Immunotherapy vaccine regimen in patients with advanced cancer	Gatti‐Mays et al. (271), NCT03384316
28	Biological: Prevnar- Pneumococcal Conjugate Vaccine (PCV) Other: Activated/costimulated autologous T-cell Drug: Revlamid^®^ (Lenalidomide) Biological: MAGE-A3/GM-GSF, Hiltonol^®^ (Poly-ICLC)	MAGE-A3	Combination Immunotherapy and Autologous Stem Cell Transplantation for Multiple Myeloma	Rapoport et al. (272), NCT01245673
22	Biological: monoclonal antibodyDrug: chemotherapy	HLA-DR	Immunostimulant Antibody in Combination with Chemotherapy for Advanced Cancer of the Pancreas	Beatty et al. (273), NCT00711191
17	Genetic: 1RG-CART Drug: Cyclophosphamide Drug: Fludarabine Other: Leukapheresis	GD2	Anti-GD2 Chimeric Antigen Receptor (CAR) Transduced T-cells (1RG-CART) in Patients with Relapsed or Refractory Neuroblastoma	Straathof et al. (274), NCT02761915
30	Drug: Cyclophosphamide Drug: Fludarabine Biological: Anti-B-cell maturation antigen (BCMA) chimeric antigen receptor (CAR) T cells	BCMA	Cells Targeting B-Cell Maturation Antigen for Previously Treated Multiple Myeloma	Abbas Ali et al. (265), NCT02215967
12	Drug: Fludarabine Drug: Cyclophosphamide Biological: E6 TCR Drug: Aldesleukin	PD-1	T Cell Receptor Gene Therapy Targeting HPV-16 E6 for HPV-Associated Cancers	Hinrichs et al. (275), NCT02280811
16	Biological: 2 vaccine injections in 1 limb Biological: 2 vaccine injections in distinct limbs Biological: 2 “vaccine injections” in distinct limbs	LAG3 MAGE-3 NA-17 NY-ESO-1	Immunotherapy of HLA-A2 Positive Stage II-IV Melanoma Patients (LAG-3/IMP321)	Legat et al. (276), NCT01308294

Sensitization of the tumor to chemotherapy is mostly connected with mitosis inhibition or induction of additional mutations in the tumor site using radiotherapy or irradiation. In this case, the tumor is less aggressive and viable, therefore the effects of additional chemotherapy are achieved more easily and fewer tumors are resistant to the therapy. However, the modulation of membrane antigens as a supportive method before chemo- or immunotherapy remains a novel idea. The first attempts at the therapy came with the use of photodynamic therapy as an adjuvant method for cancer treatment. The tumor site is irradiated with a photosensitizer after the excision of the vast majority of the tumor. The radiation involves increased infiltration of the tumor site by immune cells and thus prevents the rebuilding of the tumor from the local metastatic cells. The process is related to the increased abundance of DAMPs in the tumor site and the effect of PDT on tumor-associated immune cells. Similarly, treatment with a pulsed electric field in the irreversible electroporation protocol is associated with an increased release of DAMPs to the tumor microenvironment and the sensitization of APCs to tumor-associated molecules. However, in this case, PEF treatment also directly affects the T-cells, making them more cytotoxic toward the tumor. **Table 2** presents the currently ongoing clinical trials in this area.

**Table 2:** Active clinical trials.

Number of Patients	Therapy Protocol	Target Antigen	Short Description	Clinical Trial Registry	Trial Phase
60	Biological: Patient-derived CD19- and CD22 specific CAR	CD19, CD22	Dual Specificity CD19 and CD22 CAR-T Cell Immunotherapy for CD19+CD22+ Leukemia	NCT03330691	Phase 1
60	Biological: CAR-T Cell Combination Product: CAR-T combining PD-1 Knockout	MUC1, PD-1	CAR-T Cell Immunotherapy for Lung Cancer	NCT03525782	Phase 1 Phase 2
26	Biological: CAR-20/19-T cells	CD19, CD20	CAR-20/19-T Cells in Patients with Lymphoma and Lymphocytic Leukemia	NCT03019055	Phase 1
86	Drug: ZW25 (Zanidatamab) Drug: Palbociclib Drug: Fulvestrant	HER2+, HR+	ZW25 (Zanidatamab) With Palbociclib Plus Fulvestrant in Patients with HER2+/HR+ Advanced Breast Cancer	NCT04224272	Phase 2
18	Drug: Atezolizumab Drug: Cyclophosphamide Drug: Fludarabine Biological: MAGE**-**A1-specific T Cell Receptor-transduced Autologous T-cells Biological: PD1 Inhibitor	MAGE-A1	MAGE-A1-specific T Cell Receptor-transduced Autologous T-cells and Atezolizumab for the Treatment of Metastatic Triple Negative Breast Cancer, Urothelial Cancer and NSCLC	NCT04639245	Phase 1 Phase 2
86	Biological: ChAdOx1-MAGEA3-NYESO Biological: MVA-MAGEA3 Combination Product: Standard of care treatment	MAGE-A3, NY-ESO-1	ChAdOx1 and MVA Vaccines Against MAGE-A3 and NY-ESO-1	NCT04908111	Phase 1 Phase 2
208	Biological: CD22-CAR	CD22	Anti-CD22 Chimeric Receptor T Cells in Pediatric and Young Adults with Recurrent or Refractory CD22-expressing B Cell Malignancies	NCT02315612	Phase 1
167	Biological: Patient Derived CD19 specific CAR T cells also expressing EGFRt	CD19	Genetically Modified T Cells Directed Against CD19 for Relapsed/Refractory CD19+ Leukemia	NCT02028455	Phase 1 Phase 2
49	Drug: ADG106	CD137	CD137 Agonist ADG106 in Patients with Advanced or Metastatic Solid Tumors and/or Non-Hodgkin Lymphoma	NCT03707093	Phase 1
32	Drug: INCMGA00012 Drug: INCAGN01876 Drug: SRS Procedure: Brain surgery	GITR	Anti-GITR Agonist INCAGN1876 and the PD-1 Inhibitor INCMGA00012 in Combination with Stereotactic Radiosurgery in Recurrent Glioblastoma	NCT04225039	Phase 2

## Summary

Modulation of membrane antigens is a promising attempt in anticancer therapy. Modern oncology should not only focus on the induction of cell death in tumor cells but also aim to sensitize the body toward cancer cells and prepare the targeted tumor for anticancer therapy to increase its efficiency. The various methods that could be used to modify tumor antigens include pharmacotherapy or treatment with ultrasounds, X-rays, electric and magnetic field. Not only changes in the tumor membrane may be beneficial in the therapy – also alternations in immune system cells may result in a better therapeutic outcome.

## Author Contributions

WS, NJ, NS, and OM contributed to conception and design of the study. BN organized the database. WS, NJ, and NS wrote the first draft of the manuscript. All authors contributed to manuscript revision, read, and approved the submitted version.

## Funding

The authors would like to acknowledge the funding by the Polish Ministry of Education and Science under project number SKN/SP/496717/2021 and partial funding by the Department of Molecular and Cellular Biology under project No SUB.D260. 22.016 and the National Science Centre (Poland) project No. 2017/27/N/NZ3/01110 (to OM). The publication was prepared under the project financed from the funds granted by the Ministry of Education and Science under the “Regional Initiative of Excellence” programme for the years 2019-2022, project number 016/RID/2018/19, the amount of funding PLN 9,354,023.74.

## Conflict of Interest

The authors declare that the research was conducted in the absence of any commercial or financial relationships that could be construed as a potential conflict of interest.

## Publisher’s Note

All claims expressed in this article are solely those of the authors and do not necessarily represent those of their affiliated organizations, or those of the publisher, the editors and the reviewers. Any product that may be evaluated in this article, or claim that may be made by its manufacturer, is not guaranteed or endorsed by the publisher.
